# miRNA-Based Rapid Differentiation of Purified Neurons from hPSCs Advancestowards Quick Screening for Neuronal Disease Phenotypes In Vitro

**DOI:** 10.3390/cells9030532

**Published:** 2020-02-25

**Authors:** Mitsuru Ishikawa, Takeshi Aoyama, Shoichiro Shibata, Takefumi Sone, Hiroyuki Miyoshi, Hirotaka Watanabe, Mari Nakamura, Saori Morota, Hiroyuki Uchino, Andrew S. Yoo, Hideyuki Okano

**Affiliations:** 1 Department of Physiology, Keio University School of Medicine, 35 Shinanomachi, Shinjuku-ku, Tokyo 160-8582, Japan; ishimi@keio.jp (M.I.); marinakamura@keio.jp (M.N.); 2 Department of Anesthesiology, Tokyo Medical University, 6-7-1 Nishishinjuku, Shinjuku-ku, Tokyo 160-0023, Japan; 3 Department of Developmental Biology, Washington University School of Medicine, St. Louis, MO 63110, USA

**Keywords:** human pluripotent stem cell, excitatory neuron, neurogenin2, microRNA-9/9*, microRNA-124, Alzheimer’s disease, presenilin1, presenilin2

## Abstract

Obtaining differentiated cells with high physiological functions by an efficient, but simple and rapid differentiation method is crucial for modeling neuronal diseases in vitro using human pluripotent stem cells (hPSCs). Currently, methods involving the transient expression of one or a couple of transcription factors have been established as techniques for inducing neuronal differentiation in a rapid, single step. It has also been reported that microRNAs can function as reprogramming effectors for directly reprogramming human dermal fibroblasts to neurons. In this study, we tested the effect of adding neuronal microRNAs, miRNA-9/9*, and miR-124 (miR-9/9*-124), for the neuronal induction method of hPSCs using Tet-On-driven expression of the Neurogenin2 gene (*Ngn2*), a proneural factor. While it has been established that *Ngn2* can facilitate differentiation from pluripotent stem cells into neurons with high purity due to its neurogenic effect, a long or indefinite time is required for neuronal maturation with *Ngn2* misexpression alone. With the present method, the cells maintained a high neuronal differentiation rate while exhibiting increased gene expression of neuronal maturation markers, spontaneous calcium oscillation, and high electrical activity with network bursts as assessed by a multipoint electrode system. Moreover, when applying this method to iPSCs from Alzheimer’s disease (AD) patients with *presenilin-1* (*PS1*) or *presenilin-2* (*PS2*) mutations, cellular phenotypes such as increased amount of extracellular secretion of amyloid β42, abnormal oxygen consumption, and increased reactive oxygen species in the cells were observed in a shorter culture period than those previously reported. Therefore, it is strongly anticipated that the induction method combining *Ngn2* and miR-9/9*-124 will enable more rapid and simple screening for various types of neuronal disease phenotypes and promote drug discovery.

## 1. Introduction

Utilization of human pluripotent stem cells has become important for recapturing human development and for disease analysis that would provide a basis for regenerative medicine and drug discovery. In particular, the discovery of human induced-pluripotent stem cells (iPSCs) [1] enabled researchers to overcome the issue of direct inaccessibility to the human brain, which has been a major barrier in patient cell-based research. It is not an overstatement to say that analysis and cell therapy using neural cells differentiated from patient-derived iPS cells has increasingly become more attainable [2,3]. Meanwhile, some technical problems concerning neuronal induction still remain. First, it is necessary to increase the purity of the target neurons. Methods for inducing neural differentiation through embryoid body’s (EB’s) and neural stem cells using various physiological substances or activators/inhibitors of cellular signaling have been investigated. A previous report introduced a method that promotes neuronal differentiation through dual SMAD signal inhibition, which was proven to be very effective [4]. Subsequently, culture methods targeting various types of neuronal cells have been developed and are useful to some extent for analyzing the pathophysiology of neuronal diseases [5,6,7]. Alternatively, it has been reported that direct differentiation into neurons from hPSCs can be achieved by overexpressing Neurogenin2 (Ngn2), a bHLH-type proneural factor [8]. By manipulating Ngn2 expression with a tet-inducible expression system, all cells that have survived drug selection could be induced into excitable neurons in a doxycycline-dependent manner. As 100% differentiation/induction can be achieved in principle using this method, variability of induction efficiency between experimental batches and clones will be reduced, resulting in much higher credibility of biochemical analyses [9,10]. Although this neural cell purification technique is very useful, the extensive time required for neuronal maturation remains an issue. Thus, the use of various medium compositions and co-culture with astrocytes has been previously tested to promote neuronal maturation [11,12].

In this study, we aimed to manipulate transient gene expression and develop a method that can accelerate functional maturity while maintaining high purity induction of neurons with Ngn2 expression. Here, we focused on microRNA-9/9* (miR-9/9*) and microRNA-124 (miR-124), which have been implicated in efficient neuronal differentiation and functional maturity [13,14]. miR-9/9* and miR-124 function as a molecular switch of chromatin modifiers and antagonize anti-neurogenic pathways by suppressing USP14, leading to the destabilization of EZH2 and REST, which is then accompanied by dynamic changes in chromatin structure [15,16]. Specifically, miR-9 and miR-124 have been shown to suppress BAF53a (a subunit of the BAF chromatin remodeling complex)-induced neural progenitor proliferation, thereby enhancing the switch to the BAF53b neuron specific subunit, which strongly promotes neurite outgrowth [16,17,18,19,20]. These miRNAs have attracted attention in recent years because their overexpression can directly and efficiently transdifferentiate human adult fibroblasts to neurons [21,22]. Of note, it is assumed that cells are subjected to strong stress when undergoing intense conversion [23]; however, temporarily inhibiting caspase activity could effectively suppress programmed cell death. We believe that transiently expressing Bcl-xL, which can suppress cellular stress associated with cell fate conversion [24], will also be effective when converting iPSC into neurons. In this study, we show that the combination of the expression of *Ngn2* and Bcl-xl-miRNA-9/9*-124 leads to robust neural differentiation and maturation from pluripotent stem cells. Additionally, we describe the potential of this method as an efficient and robust system that can be used for screening neuronal pathophysiology and drug discovery.

## 2. Materials and Methods

### 2.1. Undifferentiated hPSC Culture

The hESC line KhES1 [25], the hiPSC lines 1210B2 [26], 201B7 [1], and 414C2 [27] were used as the healthy control human hPSC lines. The PS1-mutated hiPSC lines PS1-2 and the PS2-mutated hiPSC lines PS2-1 [24] were used as the FAD iPSC lines. hPSCs were cultured with or without feeder cells. For on-feeder culture, iPSCs were grown on mitomycin-Ctreated SNL murine fibroblast feeder cells in standard human pluripotent stem cell medium (DMEM/F12 medium, FUJIFILM Wako Pure Chemical, Osaka, Japan) containing 20% Knock Out Serum Replacement, KSR (Thermo Fisher Scientific, Waltham, MA, USA), 0.1 mM nonessential amino acids, 0.1 mM 2-mercaptoethanol (Merck, Darmstadt, Land Hessen, Germany), and 4 ng/ml fibroblast growth factor 2 (PeproTech) at 37 °C in an atmosphere containing 3% CO_2_. The feeder-free culture was prepared as previously described [26,28] with slight modifications. Briefly, hPSCs were maintained in StemFit/AK02N (Ajinomoto, Tokyo, Japan). Cells were passaged using 0.5× TrypLE select (Thermo Fisher Scientific, Waltham, MA, USA) in PBS(-) every 7 d and seeded at 1.5 × 10^5^ cells/well of tissue culture treated six-well plate in 1.5 or 2.0 mL medium with 1.5 µg/ml iMatrix-511 silk (Laminin-511 E8, Nippi, Tokyo, Japan) in the presence of 10 µM Y27632 (Nacalai, Kyoto, Japan) for the first day. Culture media were changed on days 1, 3, and 5.

### 2.2. PiggyBac Vector Transfection

Based on a previous study [9,10,29], Ngn2-inducible iPSCs were established using the following vectors: PB-TET-PH-lox66FRT-NEUROG2, pCMV-HyPBase-PGK-Puro and PB-CAGrtTA3G-IH. These vectors were co-transfected into dissociated iPSCs using Gene Juice Transfection Reagent (Merck, Darmstadt, Land Hessen, Germany). The transfectants were cultured in StemFit/AK02N containing 20 µM Y27632, 450 µg/ml hygromycin (FUJIFILM Wako Pure Chemical, Osaka, Japan) and 2–10 µg/ml puromycin (Merck, Darmstadt, Land Hessen, Germany) on 3.0 µg/ml iMatrix-511 silk-coated tissue culture plates. Of the surviving cells, only the clones capable of neuronal induction in a Dox-dependent manner while maintaining undifferentiated properties in hPSC medium were expanded and cryopreserved for this study.

### 2.3. Preparation and Infection of Lentiviruses

Lentiviruses were purchased from VectorBuilder (Chicago, IL, USA) or produced in HEK293T cells via the transient transfection of three plasmids: the packaging construct pCAG-HIVgp, VSV-G and Rev-expressing construct pCMV-VSV-G-RSV-Rev, and self-inactivating (SIN) lentiviral vector construct (CSIV-124-9-BclxL-TRE-EF-KT, CSIV-124-9-BclxL-TRE-EF-BsdT, pLV-Puro-TRE3G-BclxL, pLV-Puro-TRE3G-Pri-miR-9-3, pLV-Puro-TRE3G-Pri-miR-124a-2, pLV-Puro-TRE3G-Pri-miR-9-3-Pri -miR-124a-2, pLV-Puro-TRE3G-BclxL-Pri-miR-9-3-Pri-miR-124a-2 and pLV-SYN1-jGCaMP7s-P2A -NLS-mCherry). As the original tet-inducible BclxL and miR-9/9*-124 lentiviral vector, pTight-9/9*-124-BclxL [24], was a second-generation lentiviral vector, we transferred the expression cassette into a third-generation self-inactivating lentiviral vector backbone. The tet-inducible all-in-one self-inactivation lentiviral vector expressing BclxL and miR-9/9*-124, CSIV-124-9/9*-BclxL-TRE-EF-KT, was constructed by a Multisite Gateway-based method as previously described [30]. The human BclxL gene or the miR-9/9*-124 was amplified by PCR by using a primer set with corresponding additional attB-signals at 5’-ends and pTight-9/9*-124-BclxL as a common template. The resultant attB-signal flanked PCR fragment, B3-hBclxL-B2 or B2r-miR-9/9*-124-B4 was cloned into pDONR-P3P2 or pDONR-P2rP4 [30], respectively, by Gateway BP cloning. The resultant entry clones, pENTR-B3-hBclxL-B2 and pENTR-B2r-miR-9/9*-124-B4, were assembled into a tet-inducible Multisite Gateway-compatible self-inactivation lentiviral vector, CSIV-DEST-R4R3-TRE-EF-KT, by Gateway LR cloning. CSIV-DEST-R4R3-TRE-EF-KT was a derivative of a tet-inducible Gateway-compatible self-inactivation lentiviral vector, CSIV-RfA-TRE-EF-KT [31], the attR1 and attR2 signals of which were converted into attR3 and attR4 signals, respectively, for enabling Multisite Gateway LR cloning by BP reaction with a modified conversion vector [32]. The conversion vector, pENTR-L1-R3-TetR-ccdB-R4-L2, was modified from the original pDONR-R3-R4 vector, in order to obtain a tetracycline-resistance gene instead of a chloramphenicol-resistance gene to facilitate selection of the converted Multisite Gateway vector. A slightly modified vector, CSIV-124-9/9*-BclxL-TRE-EF-BsdT, in which a humanized monomeric Kusabira-Orange 1 (mKO1) fluorescent protein gene [33] is replaced by the blasticidin-resistance gene, was also constructed by replacing the GFP expression cassette of CSIV-GFP-TRE-EF-BsdT with the expression cassette of CSIV-124-9/9*-BclxL-TRE-EF-KT.

For generating lentiviral vectors, on day 0, HEK293T cells were passaged in Poly-ornithine-coated dishes in DMEM containing 10% FBS and cultured at 37 °C and 5% CO_2_ for 24 h. The transfection of each gene and packaging and coat proteins were performed using polyethylenimine (PEI). For each dish, a cocktail of SIN, pCAG-HIVgp, pCMV-VSV-G-RSV-Rev, and PEI was suspended in Opti-MEM (Thermo Fisher Scientific, Waltham, MA, USA). Subsequently, the DNA/PEI mixture was added to the cells and after 12 h, the supernatant was changed to DMEM with 3% FBS containing 10 μM forskolin to enhance virus production. After 48 h, the supernatant was collected and filtered through a 0.45 μm filter (Millipore, Burlington, MA, USA). Lentiviruses were resuspended with Opti-MEM or PBS (-) and frozen at −80 °C until further analysis. For infection, lentiviral solutions were added to hPSCs with MOI = 2.0, followed by incubation for 48 h, after which the total hPSCs medium was changed with fresh medium. 

### 2.4. Neuronal Differentiation by Transient Expression of Ngn2 and/or microRNAs from hPSCs

Feeder-free cultured hPSCs were used for the induction of neuronal differentiation. The cells were dissociated using 0.5× TrypLE Select and seeded on culture dishes coated with poly-L-lysine, and iMatrix-511. To induce glutamatergic neurons, Ngn2-transduced cells were cultured in neuronal medium with the following composition: Neurobasal Plus medium containing 2% B27 Plus supplement, 1% Culture One supplement, 1% Glutamax (Thermo Fisher Scientific, Waltham, MA, USA), 200 µM L-ascorbic acid (Merk, Darmstadt, Land Hessen, Germany), 100 µM dbcAMP (Nacalai, Kyoto, Japan), 20 ng/ml BDNF, 20 ng/ml GDNF, 20 ng/ml NT-3 (Alomone labs, Jerusalem, Israel) and 0.5 µg/mL iMatrix-511 silk. 20 µM Y27632 and 2 µg/ml Dox (FUJIFILM Wako Pure Chemical, Osaka, Japan) were added on day 0. On day 5, the medium was replaced with the fresh neuronal medium described above. For the CellROX assay and extracellular flux analyses assay, cells were maintained in the following medium after day 5: Neurobasal plus medium containing 2% B27 minus antioxidants, 1% Culture One supplement, 1% Glutamax, 20 ng/ml BDNF, 20 ng/ml GDNF, 200 µM L-ascorbic acid, 300 µM dbcAMP and 0.5 µg/ml iMatrix-511 silk. The medium was replaced every 3 days after day 5.

### 2.5. Neuronal Differentiation Using Dual SMAD Inhibition from hPSCs

We modified the protocol for neuronal induction shown in [7]. Briefly, for preparing dual SMAD inhibitor-treated hPSCs, cells were cultured in Stem Fit/AK02N containing 1 µM SB431542 (Merck, Darmstadt, Land Hessen, Germany), 1 µM Dorsomorphin (Santa Cruz Biotechnology, Dallas, TX, USA) and 1 µg/ml Y27632 from day 1 to 7. On day 7, cells were dissociated into single cells using TrypLE Select and cultured in KBM Neural Stem Cell (KOHJIN BIO, Sakado, Saitama, Japan) containing B27 supplement with 2 µM SB431542, 2 µM IWP-2 (Merck, Darmstadt, Land Hessen, Germany) and 10 µM Y27632 at 4% O_2_/5% CO_2_. On day 14, cells were dissociated again, followed by culture in the same conditions as day 7. On day 20, cells were dissociated into single cells using TrypLE Select and plated on culture plates coated with Matrigel (Corning, Steuben County, NY, USA) and cultured in a neuronal medium (Neurobasal plus medium containing 2% B27 plus supplement, 1% Culture One supplement, 1% Glutamax, 200 µM L-ascorbic acid, 300 µM dbcAMP, 20 ng/ml BDNF, 20 ng/ml GDNF, 20 ng/ml NT-3 and 0.5 µg/ml iMatrix-511) at the normal (20%) O_2_ concentration/5% CO_2_. The medium was replaced every 3 days.

### 2.6. RNA Extraction and Quantitative Reverse-Transcription PCR

RNA was extracted from the cells using the microRNeasy mini Kit (Qiagen, Hilden, North Rhine-Westphalia, Germany). One hundred nanograms of RNA was used to prepare cDNA using the iScript cDNA Synthesis Kit (Bio-Rad, Hercules, CA, USA). qRT-PCR was performed with SYBR Premix ExTaqII (TaKaRa Bio, Kusatsu, Shiga, Japan) using the ViiA 7 real-time PCR system (Thermo Fisher Scientific, Waltham, MA, USA). Values were normalized to ActB levels. The comparative (ΔΔCt) method was used to analyze the data. See the sequences of primers used for qRT-PCR in Supplemental Table S1.

### 2.7. Immunofluorescence Analysis

Cells were fixed with 4% paraformaldehyde for 20 min at room temperature and then incubated with blocking buffer (PBS(-) containing 2% NGS, 2% BSA, and 0.2% Triton X-100) for 1 h at room temperature. Then, the cells were incubated overnight at 4°C with primary antibodies (see Supplemental Table S2), which were diluted in blocking buffer without Triton X-100. The cells were washed three times with PBS and then incubated with secondary antibodies Alexa Fluor 488, Alexa Fluor 555, or Alexa Fluor 647 (1:1000; Thermo Fisher Scientific, Waltham, MA, USA) and 10 µg/ml Hoechst 33342 (Merck, Darmstadt, Land Hessen, Germany) for 1h at room temperature. After washing with PBS, the cells were examined with a BZ-9000 microscope (Keyence, Osaka, Japan), IN Cell Analyzer 6000 (GE Healthcare, Chicago, IL, USA) and LSM700 (Zeiss, Oberkochen, Land Baden-Württemberg, Germany). The primary antibodies used in this study are listed in Supplemental Table S2.

### 2.8. High-Content Image Analysis

For cell population assays, stained plates were imaged with an IN Cell Analyzer 6000; a set of 5 × 5 fields was collected from each well using the 20× objective lens. For quantitative analysis of the synaptic area, stained plates were also imaged with an IN Cell Analyzer 6000; a set of 6 × 6 fields was collected from each well using the 60× objective lens. Analysis was performed using IN Cell Developer Toolbox version 1.9 (GE Healthcare, Chicago, IL, USA). Intact nuclei stained by the Hoechst dye were identified and those that were >50 µm^2^ in surface area and with intensity levels that were typical and lower than the threshold brightness of pyknotic cells were defined as traced nuclei. Each traced nuclear region was then expanded by 50%. Using the expanded nuclear region images, neuronal markers (Tubb3, MAP2, and NeuN) were identified and the percentage of each marker was calculated from these images. Using these traced neuronal images of each cell, neurite length was analyzed. Synaptic areas were defined by binarized images of Synapsin I or PSD95, which overlapped with the Tubb3-stained area.

### 2.9. Calcium Imaging

hPSCs were infected with jGCaMP7s lentivirus (MOI = 2) 6 days before neuronal induction for calcium imaging. Calcium imaging analyses were performed on day 20 after neuronal induction on a glass-bottom 96-well multi plate (Eppendorf, Hamburg, Germany). Half of the culture medium was replaced, following 10 µg/ml Hoechst treatment 24 h before imaging. Changes in fluorescence intensity were measured using an IX83 inverted microscope (Olympus, Tokyo, Japan) equipped with an Electron Multiplying CCD Camera (Hamamatsu Photonics, Hamamatsu, Shizuoka, Japan) and LED illumination system pE-4000 (CoolLED, Andover SP10 5NY, UK). We recorded 5000 frames (1 frame: 31–32 ms) per well using the stream acquisition mode. MetaMorph Image Analysis Software (Molecular Devices, San Jose, CA, USA) was used to analyze the live cell calcium traces. Regions of interests (ROIs) were set only for mCherry-positive neurons. ROIs were drawn on cells based on time projection images of the recordings. ROI traces of the time course of changes in fluorescence intensity were generated and used as substrates for subsequent analyses. To adjust for photobleaching, the difference in intensity between the first and last frames was calculated and subtracted from the raw intensity. The change in fluorescence intensity over time was normalized as ΔF/F= (F-F0)/F0, where F0 is fluorescence at the starting point of exposure (time 0). ΔFmax was defined as the difference of the largest change in ΔF/F.

### 2.10. Multiple/Microelectrode Array (MEA) Analysis

MEA was assayed using the Maestro system (Axion Biosystems, Atlanta, GA, USA). Neuronal inductions from hPSCs were performed in 48-well MEA plates coated with polyethylene imine and 50× Matrigel (Corning). A total 1.5 × 10^5^ cells in 30 µl culture medium were seeded in an electrode well. Thirty minutes after seeding, 470 µl of culture medium was added. For neuronal differentiation, hPSCs were cultured in induction medium for the first 5 days and then in maintenance medium until day 35. Data were acquired using a sampling rate of 12.5 kHz and filtered using a 200–3000 Hz Butterworth bandpass filter. The detection threshold was set to +6.0 × SD of the baseline electrode noise. Five minutes of activity were subsequently recorded at 37 °C. Given the considerable variation in spike count between wells and plates, we focused on active electrodes (electrodes with an average of >5 spikes/min), which were certain to make contact with neurons. The number of active electrodes and the mean spike rate per active electrode (spikes per second) was calculated using the spike count file generated by the Axion Integrated Studio program (Axion Biosystems, Atlanta, GA, USA). The spike raster plots were generated using the Neural Metric Tool (Axion Biosystems, Atlanta, GA, USA). Single electrode bursts were defined as the count number of >5 spikes/100 ms. Network bursts were defined as the count number of >50 spikes/100 ms occurring simultaneously in 35% of the electrodes.

### 2.11. ELISA for Aβs

iPSCs were seeded at a density of 1.0 × 10^5^ cells/well in a 48-well plate and cultured in neuronal medium with 2 µg/ml Dox and 2 µg/ml Y27632 at 37 °C in 5% CO_2_. Five days after seeding the cells, the medium was replaced with fresh neuronal medium, and cells were cultured until day 20 or day 40. The culture medium was fully changed with 500 µl/well of fresh medium 72 h before the harvest. The culture supernatant was centrifuged at 200× *g* to remove insoluble material and the supernatants were collected and stored at −80 °C. The remaining neuronal cells were lysed in RIPA buffer (Santa Cruz Biotechnology, Dallas, TX, USA), and the protein concentration was measured by BCA Protein assay (Thermo Fisher Scientific, Waltham, MA, USA). Aβ40 and Aβ42 levels in the conditioned medium were measured using the Human β Amyloid (1–40) ELISA Kit II (FUJIFILM Wako Pure Chemical, #298–64601, Osaka, Japan) and Human β Amyloid (1–42) ELISA Kit High Sensitive (FUJIFILM Wako Pure Chemical, #296–64401, Osaka, Japan), according to the manufacturer’s protocol. The concentration of each Aβ species was normalized by the protein levels in the culture.

### 2.12. Determination of OCRs

Mitochondrial OCRs in cell cultures were measured using an XFe24 Extracellular Flux Analyzer (Agilent Technologies, Santa Clara CA, USA). The iPSCs were seeded at a density of 5 × 10^4^ cells/well in a XFe24-well cell culture microplate (Agilent Technologies, Santa Clara CA, USA) and cultured in neuronal medium with doxycycline at 37 °C in a 5% CO_2_ atmosphere. Five days after seeding the cells, the medium was replaced with culture medium without doxycycline, and the cells were cultured for 7 or 15 days. The medium was replaced with XF DMEM medium (Agilent Technologies, Santa Clara CA, USA) containing 10 mM glucose, 1 mM sodium pyruvate, and 2 mM L-glutamine, and the plates were pre-incubated in a CO_2_-free incubator at 37 °C for 1 hour for equilibration and thereafter processed in the XF analyzer for OCR analysis. OCRs were recorded three times, followed by sequential injections of 1 µM oligomycin, 0.5 µM FCCP, and 0.5 µM antimycin A and rotenone into each well. The basal OCR was calculated by subtracting the OCR values obtained after the addition of rotenone and antimycin A from the OCR values of the third measurement of the experiment. The spare respiratory capacity was calculated using the ratio of maximal OCR, which was calculated by subtracting the OCR values obtained after the addition of rotenone and antimycin A from the OCR values obtained after the addition of FCCP, against basal OCR. As it is difficult to perform the assay in many clones, we selected one clone each for 414C2, 1210B2, PS1-2 and PS2-1 to effectively perform the assay. After the analysis, cells were fixed with 4% paraformaldehyde for 20 min at room temperature and stained with 10 µg/ml Hoechst (Ho). Data were normalized by the number of Ho-positive cells.

### 2.13. Analysis of Antioxidant Activities

Oxidant levels in neurons were estimated using CellROX Green (Thermo Fisher Scientific, Waltham, MA, USA), a fluorogenic probe for measuring oxidative stress in live cells. Neurons were incubated with 5 μM CellROX for 30 min at 37 °C in the dark and washed once with PBS. Cells were fixed with 4% PFA and subjected to immunocytochemical analyses using the anti-Tubb3 antibody. Tubb3+ cells or Tubb3+/CellROX+ cells were counted using IN Cell Analyzer 6000. Percentages of CellROX+ neurons were defined as the number of Tubb3+/CellROX+ cells divided by the number of Tubb3+ cells.

### 2.14. Statistics

Statistical analyses were performed by Dunnett’s or Tukey’s test for multiple comparison, or Student’s t-test for single comparison. Probability values (*p* value) < 0.05 were considered to be statistically significant.

## 3. Results

### 3.1. Acquisition of Neural Differentiation Ability by Overexpression of miRNA-9/9 * and miR-124 for hPSCs

**Figure 1:**
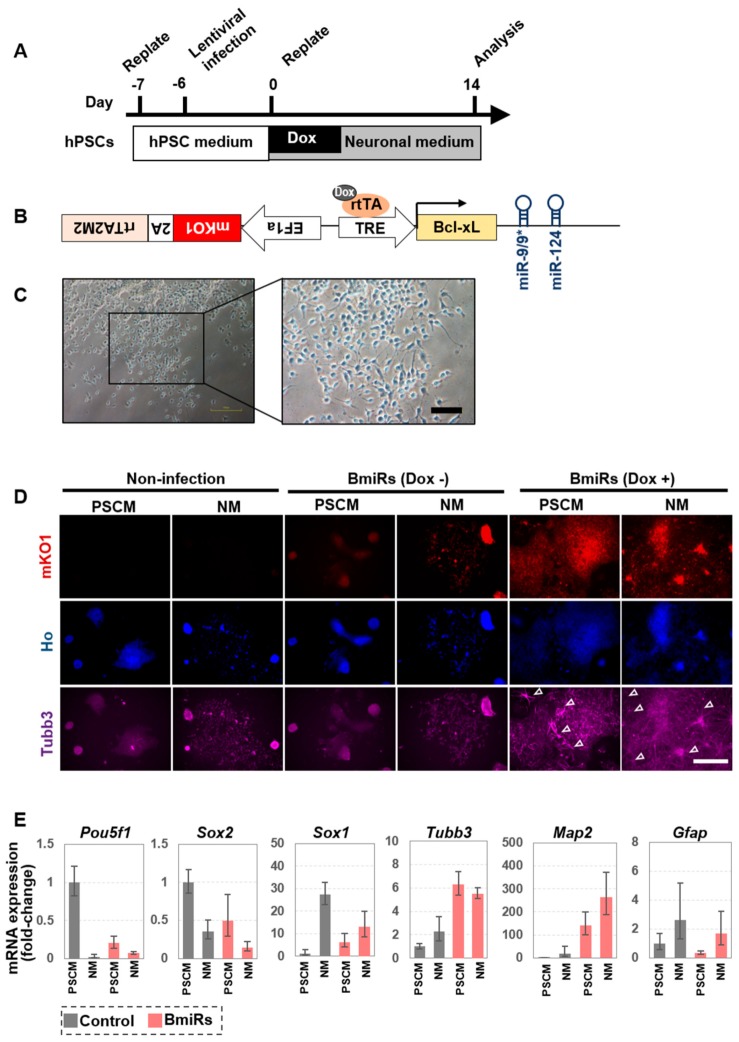
Bcl-xL genes (BmiRs) expression alone promotes neural differentiation from human pluripotent stem cells (hPSCs). (**A**) Overview of the protocol for differentiation into neurons by Tet-On-inducible Bcl-xL gene miR9/9*-124 overexpression. (**B**) Design of the lentiviral vector for Bcl-xL-miR-9/9*-124-mediated conversion of hESCs to neuronal cells. mKO1 and rtTA2M2 are abbreviations for monomeric Kusabira Orange, a fluorescent protein, and reverse tetracycline transactivator, respectively. (**C**) Representative bright field images illustrating the neuronally converted KhES1 cells at day 14. Scale bar: 100 µm. (**D**) Immunofluorescent (Tubb3) images of KhES1 in three different culture conditions: neuronal medium without Dox; hPSC medium or neuronal medium with 2 µg/ml Dox at day 20. Arrowheads indicate Tubb3+ neurite morphology. Scale bar: 500 µm. (**E**) Relative mRNA expression levels of *Pou5f1*, *Sox2*, *Sox1*, *Tubb3*, *Map2,* and *Gfap* in KhES1. (PSM: medium for pluripotent stem cells; NM: medium for neurons). Each data was standardized by the control cells cultured in PSM. (n = 4 independent experiments; mean ± SEM).

Among various PSC culture methods, we decided to use StemFit/AK02N (Ajinomoto) as the culture medium, since PSCs rapidly grow in this medium from a single cell level, and Laminin511 E8 fragment (iMatrix-511, Nippi) as the adhesion substrate [26,28]. The Neurobasal B27 Plus system was used as a medium for differentiation and maintenance of the neuronal cells (Figure 1A). We first examined whether miR-9/9*-124 have intrinsically positive effects on neuronal differentiation. The lentiviral vector used here consists of a sequence in which miR-9/9* and miR-124 are both linked downstream of the Bcl-xL gene and are driven by the TetO promotor (named BmiRs hereonafter). Furthermore, mKO1, a fluorescent protein, and rtTA were placed in the opposite direction (Figure 1B). First, KhES1, a human ES cell line [25], was maintained and cultured in a feeder-free PSCs culture system [28]. One day after the passage, the lentivirus was infected at MOI = 2, and 2 days later, the medium was replaced to withdraw the virus. On day 7, PSCs were dissociated into single cells and replated in neuronal medium containing Dox. Expression of mKO1 in the cells indicated successful infection of the lentivirus. Fourteen or twenty days after the induction, many, but not all, BmiRs (Dox+) cells showed Tubb3-positive neurite-like structures, while both non-infected cells and BmiRs (Dox-) cells did not show a neuron-like morphology despite the presence of the neuronal medium. Compared to the BmiRs (Dox-) cells group, the BmiRs (Dox+) cells group seemed to have accumulated mKO1 protein, probably because it entered the post-mitotic phase, and exhibited a rather strong fluorescent signal (Figure 1C,D, Figure S1). Cells were harvested and examined for neuronal gene and pluripotency-marker expressions. In the miRNA-driven group, the expression levels of neuronal marker genes were increased, while the expression of Pou5f1 decreased compared with the non-driven group. The expression of Gfap, an astrocyte marker, was slightly increased by the effect of the neuronal medium but was not changed drastically by BmiRs expression (Figure 1E). In summary, we demonstrated that the transient expression of BmiRs in a single-cell feeder-free culture system promoted differentiation into neuron-like cells, although these cells did not necessarily adopt neurite morphology. Thus, we speculated that the effects of BmiRs alone on neuronal induction might be limited.

### 3.2. Effect of BmiRs Addition on Neural Induction Using Ngn2

**Figure 2:**
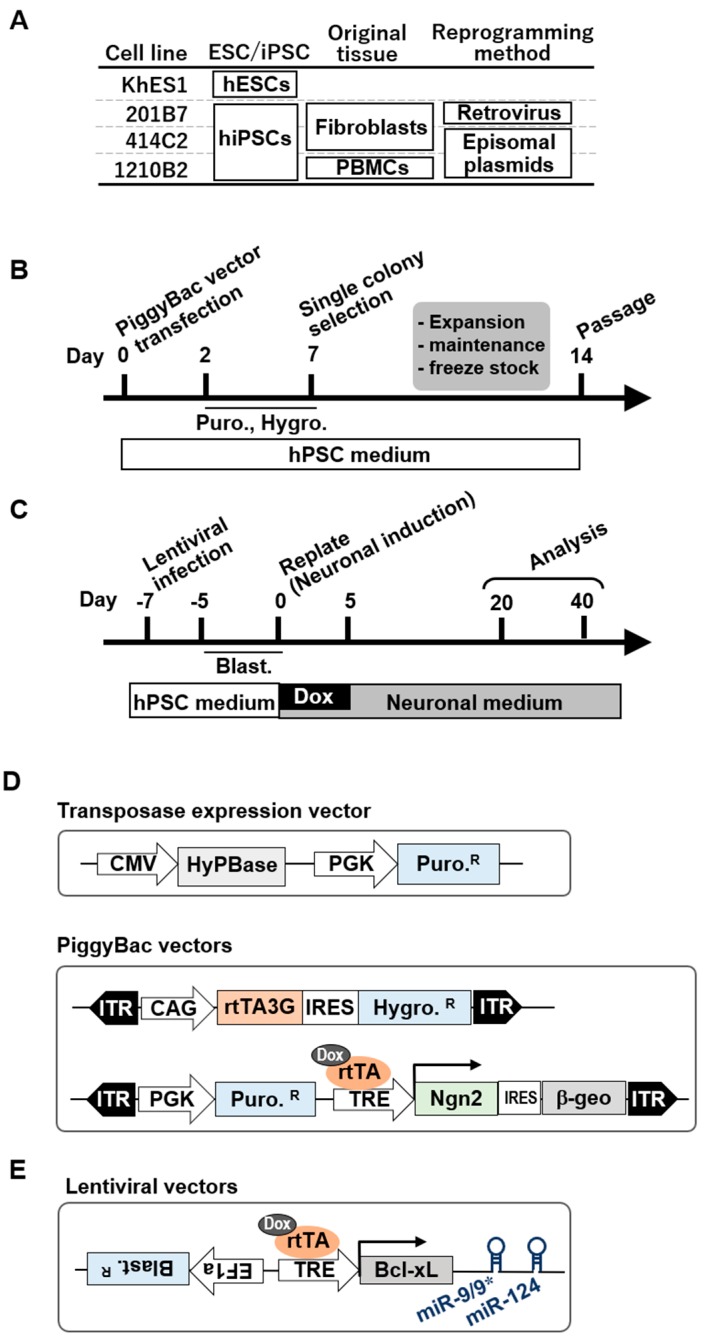

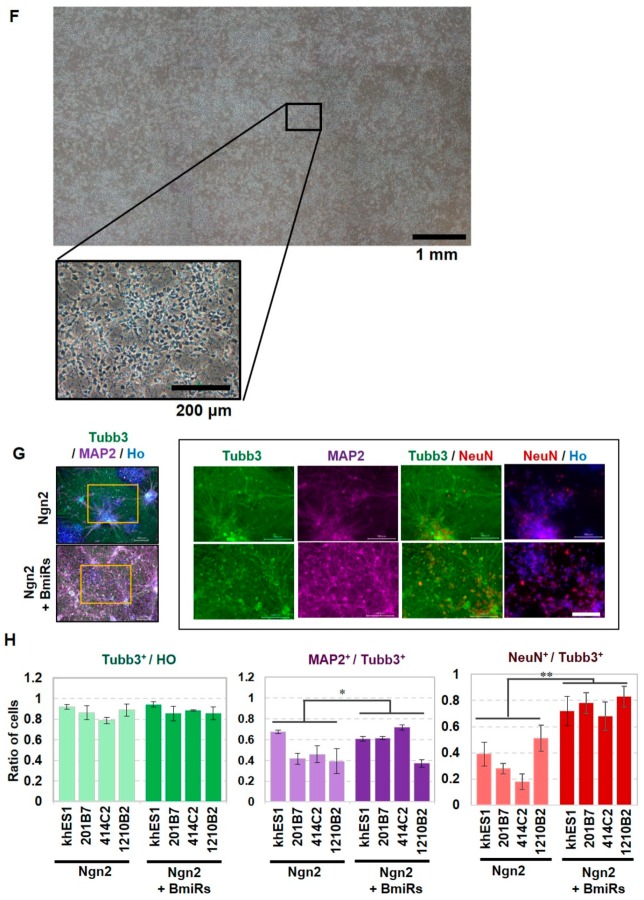
The effect of the combination of Ngn2 and BmiRs expression on neural differentiation. (**A**) The list of control hPSC line used in this study. (**B**) Overview of the protocol for piggybac vector transfection into hPSCs with the piggybac vectors. (**C**) Overview of the protocol for lentiviral infection into hPSCs and neuronal induction using Dox. (**D**,**E**) Design of the transposase expression vectors: Piggybac vectors for Ngn2-mediated neuronal conversion and lentiviral vector for Bcl-xL-miR-9/9*-124 -mediated neuronal conversion. Puro.R, Hygro.R, Blast.R, and rtTA3G indicates puromycin, hygromycin, blastcidin resistant sequences, and 3rd generation reverse tetracycline transactivator, respectively. (**F**) Representative low-power field (upper) and high-power field (lower) of the neurons (khES1) 20 days after transient expression of both Ngn2 and BmiRs. (**G**) Immunocytochemical analysis of neuronal markers (Tubb3, MAP2 and NeuN). The photograph on the right is an enlarged view of the part surrounded by yellow on the left. Scale Bar: 100 µm. (**H**) Ratio of positive cells for each marker: Tubb3^+^ cells/all cells (Tubb3+/Ho); MAP2^+^ cells/ neurons (MAP2+/Tubb3+); NeuN+ cells / neurons (NeuN+/Tubb3+) (n = 4 independent experiments; mean ± SD; * *p* < 0.05; ** *p* < 0.01, Dunnett’s test).

Next, we examined the effect of adding BmiRs for neuronal induction when using the PiggyBac vector-based Tet-On-Ngn2 gene expression system, which we had already established in previous studies [9,10]. Several types of pluripotent stem cell lines from various origins generated using multiple reprogramming methods were prepared to confirm that the results did not change despite the different background. We used KhES1 [25], a human ES cell line, 201B7 [1], which was established from human dermal fibroblasts by retroviral infection, 414C2 [27], which was established by episomal plasmids from a donor related to that of 201B7, and 1210B2 [26], which was established using episomal plasmids from peripheral blood mononuclear cells (Figure 2A). First, Tet-On-Ngn2 was introduced by the PiggyBac transposon (as seen in Figure 2D), and subsequently, drug selection was performed and hPSCs with high neuronal differentiation efficiency were subcloned (Figure 2B). Then, the lentivirus for miR-9/9*-124 expression (as seen in Figure 2E) was infected in the same manner as described above, and further drug selection was performed to obtain cells transfected with TetO-BmiRs and TetO-Ngn2. In this state, maintenance, growth, and cryopreservation of the cells were possible. Then, after single cell dissociation, differentiation was induced by Dox and the neuronal medium (Figure 2C). As a result, as in the case of the induction using Ngn2 alone, all of the stem cell lines exhibited extremely high efficiency of neural differentiation and uniform-looking neurons appeared as of day 20 (Figure 2F). In the BmiRs expression group, it was difficult to determine the neural differentiation efficiency with only brightfield imaging (Figure 2E, Figure S2A). Thus, we performed immunocytochemistry with neuronal markers. The Tubb3+ cell rate did not significantly change between the BmiRs- and BmiRs+ groups, while the MAP2+ and NeuN+ cell rates, which indicate neuronal maturation, were significantly higher in the BmiRs+ group than in the BmiRs- group (Figure 2G,H). Moreover, Ki67+ proliferating cells remained in the Ngn2-BmiRs+ group, while nearly all of the Ki67 signals disappeared by induction with Ngn2 (Figure S2B).

### 3.3. Alteration of Marker Gene Expression Levels by the Combination of Ngn2 and BmiRs

**Figure 3:**
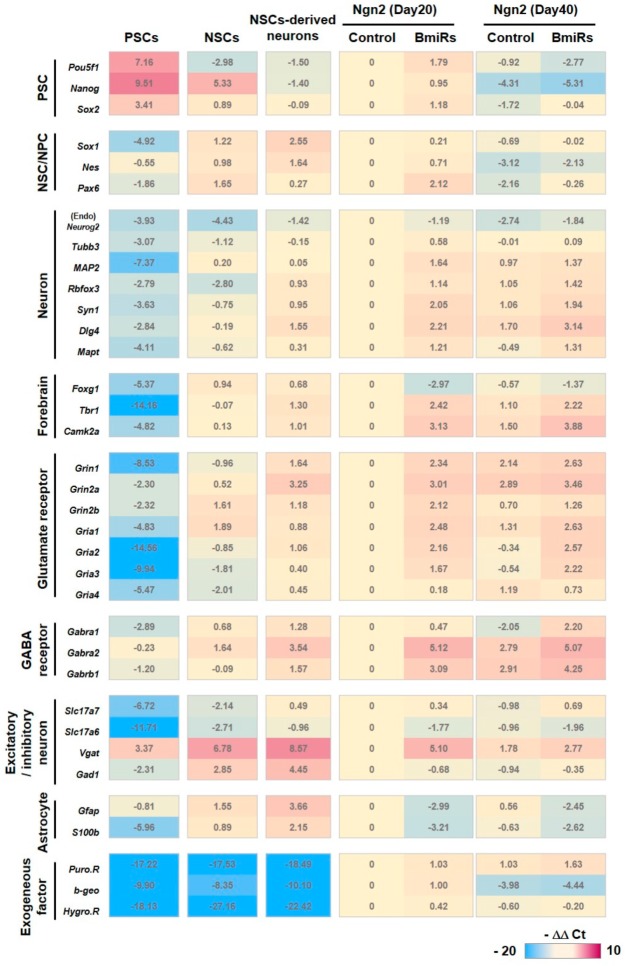
Gene expression profiling of the differentiated neurons. Using the KhES1, 201B7, 414C2, and 1210B2 lines, cells in each indicated differentiated state (PSCs, NSCs (day 20), NSCs-derived neurons (day 40) and *Ngn2*-induced neurons with or without BmiRs expression (day 20 and 40)) were harvested and subjected to Real-time RT-PCR. Analyzed data were summarized asa heatmap and corresponds to the -ΔΔCt value. The numerical value in each cell is the averaged -ΔΔCt value in each target, which is standardized by the data of *Ngn2* without BmiRs-induced neurons (Day 20).

Our results suggested that the addition of BmiRs has a positive effect on neural differentiation. Thus, quantitative PCR was performed to verify whether there were changes in specific functional properties (Figure 3). The four pluripotent stem cell lines described above were used for this experiment. For each strain, we prepared neural stem cells (NSCs) in the form of neurospheres on day 20 and terminally differentiated neurons 20 days after adhesion (i.e., 40 days in total) (as seen in Figure S3A,B), both of which were obtained using the dual SMAD inhibition method, and day 20 and 40 cells obtained by Ngn2 induction with or without BmiRs (Figure S3C).

PSC markers were apparently lower in the “Ngn2+BmiRs-(Day20)” group than in the “PSCs” group. The addition of BmiRs or long-term culture changed the PSC expression levels. NSC/NPC marker expression was lower in the “Ngn2+BmiRs- (Day20)” group compared to the NSC-derived neurons and the “BmiRs+” group showed a similar tendency. Interestingly, Pax6 tended to rise when BmiRs were added during the early culture period. It remains unclear whether this was due to the function of miRs or Bcl-xL. Neuronal gene expression in the “Ngn2+BmiRs- (Day20)” group tended to be comparable or slightly lower than that in the NSC-derived neurons. This may be because NSC-derived neurons were cultured for a total of 40 days. Conversely, in the “Ngn2+BmiRs+(Day20)” group, the expression levels of many neuronal genes were increased, particularly the synapse-related genes SynI (Synapsin I) and Dlg4 (PSD95). Forebrain-related gene expression levels tended to decrease slightly in the “Ngn2+” groups compared to the NSC-derived neurons, although curiously, their levels increased in the “BmiRs+” groups. Exceptionally, Foxg1 was reduced in the “BmiRs+” groups, possibly because miR-9/9*-124 expression might affect the expression period of Foxg1 to some extent during development. The alterations of ionotropic glutamate and GABA receptor gene expression levels were similar to those of the neuronal genes mentioned above. These gene expression levels in the “BmiRs-” groups tended to be lower than in the NSC-derived neurons, while most of them increased in the “BmiRs+” group. Thus, it is plausible that the addition of BmiRs enabled the generation of cells that are electrically functional. Regarding the excitatory/inhibitory neuronal markers, excitatory neuron marker (Slc17a7: VGluT1; Slc17a6: VGluT2) gene expression levels were not significantly different between the “Ngn2+” groups and the NSC-derived neurons, while the expression of Vgat and Gad1: Gadd67 were extremely low in the “Ngn2+” groups. For the majority of the data, this tendency did not significantly change with the addition of BmiRs or long-term culture, but interestingly, Vgat was transiently upregulated on day 20. Regarding the astrocyte-related gene expression levels, after the medium of the PSCs were switched from PSC medium to neuronal medium, the glial marker genes tended to increase somewhat (as seen in Figure 1E). The introduction of Ngn2 expression here suppressed the expression of glial marker genes (as observed when comparing the “PSCs” group and “Ngn2-BmiRs- (Day20)” group), and they were especially reduced in the BmiRs+ group. Among the exogeneous factors, genes that were constantly expressed were higher in both the early and late stages of differentiation than those in the non-transfected cell groups. Even after adding BmiRs+, their expressions altered only slightly. β-geo was specifically expressed at the same time with Ngn2 induction and its expression may have temporarily persisted until day 20. However, its expression level was sufficiently reduced by day 40 after the removal of Dox. In summary, our results indicated that the addition of BmiRs to Ngn2 enhanced the expression levels of functional genes related to neuronal differentiation and functional activity.

### 3.4. Higher Neuronal Function Acquired with the Addition of Ngn2 + BmiRs

**Figure 4:**
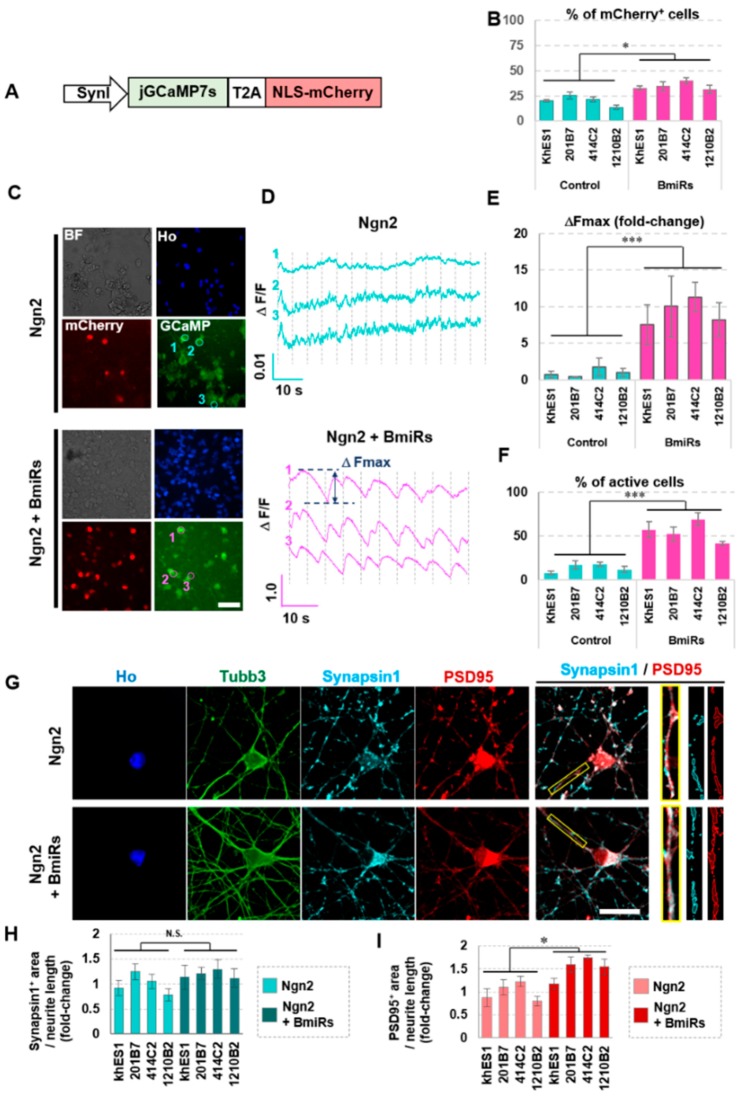
Evaluation of neuronal functionality using GCaMP and quantification of synapse-related proteins. (**A**) Design of the lentiviral vector for Synapsin1: jGCaMP7s-T2A-NLS -mCherry. (**B**) Ratio of positive cells for Synapsin1: mCherry^+^ cells in live cells. The number of total cells was defined as the total number of Ho signal. (n = 4 independent experiments; mean ± SEM; * *p* < 0.05, Dunnett’s test). (**C**) Representative GCaMP7s and mCherry images. The circled ROIs are depicted in (**D**) as time-dependent changes in GFP fluorescence intensity. Scale bar: 50 µm. (**D**) Representative images of GCaMP spikes and display of parameters (ΔFmax). (**E**) Comparison of ΔFmax values. The average value of the control group (BmiRs^−^) was used as the reference value. (n = 4 independent experiments; mean ± SD; *** *p* < 0.001, Dunnett’s test) (**F**) Ratio of calcium spike+ cells. The number of cells having a ΔFmax value of more than 0.01 in (**D**) was quantified. (n = 4 independent experiments; mean ± SEM; *** *p* < 0.001, Dunnett’s test) (**G**) Representative confocal microscope images of immunocytochemical analyses of neuronal markers and synaptic markers using confocal microscope (Scale bar: 10 µm). The enlargement of the area enclosed by the yellow rectangle in the merge photograph are shown on the right. (H,I) Comparison of SynapsinI+ (**H**) and PSD95+ (I) area colocalized with Tubb3+ area per Tubb3^+^ neurite length. The average value of the control group (BmiRs-) was used as the reference value. (n = 4 independent experiments; mean ± SEM; * *p* < 0.05, Dunnett’s test).

Studies on cell morphology and marker gene expression have shown that the combination of Ngn2 + BmiRs may promote rapid neural differentiation and maturation. To evaluate whether the neurons were functionally mature, we examined their calcium activity. To measure calcium concentration in a neuron-specific and highly sensitive manner, jGCaMP7s [25], expressed downstream of a SynI promoter, were transfected into iPSCs using lentivirus. All GCaMP-expressing cells expressed mCherry in their nuclei (Figure 4A). First, the percentage of mCherry+ cells in day 20 neurons was calculated. The percentage was higher in the Ngn2 + BmiRs group, suggesting that this group had a higher rate of differentiation and maturation (Figure 4B). We decided to only analyze the cells where GCaMP and mCherry exhibited an intensity exceeding a certain threshold. The results showed that the BmiRs group clearly showed a strong spontaneous signal oscillation with a larger population of active cells (Figure 4C–F). Interestingly, the amplitude of the signal was synchronized among different cells in the same field of view, suggesting the formation of a neural network. Then, to obtain morphological data on synapse formation, we obtained immunofluorescent images using anti-Synapsin1, a presynaptic marker and anti-PSD95, a post-synaptic scaffold protein. Confocal microscopy showed that in the Ngn2 + BmiRs group, the area of Synapsin1 per neurite did not significantly alter, while SynI mRNA expression levels were increased in Ngn2+BmiRs (Figure 3). However, the PSD95-positive area was increased (Figure 4G,H,I). Although we have yet to address why PSD95 alone makes such alteration at the protein level, it is likely that this has stabilized the synaptic junction as a result. Thus, the synchronous oscillation of GCaMP fluorescence may have occurred due to synapse formation between the neurons in the Ngn2 + BmiRs group.

### 3.5. Improvement of Neuronal Activity in Ngn2 + BmiRs Neurons as Indicated by MEA

**Figure 5:**
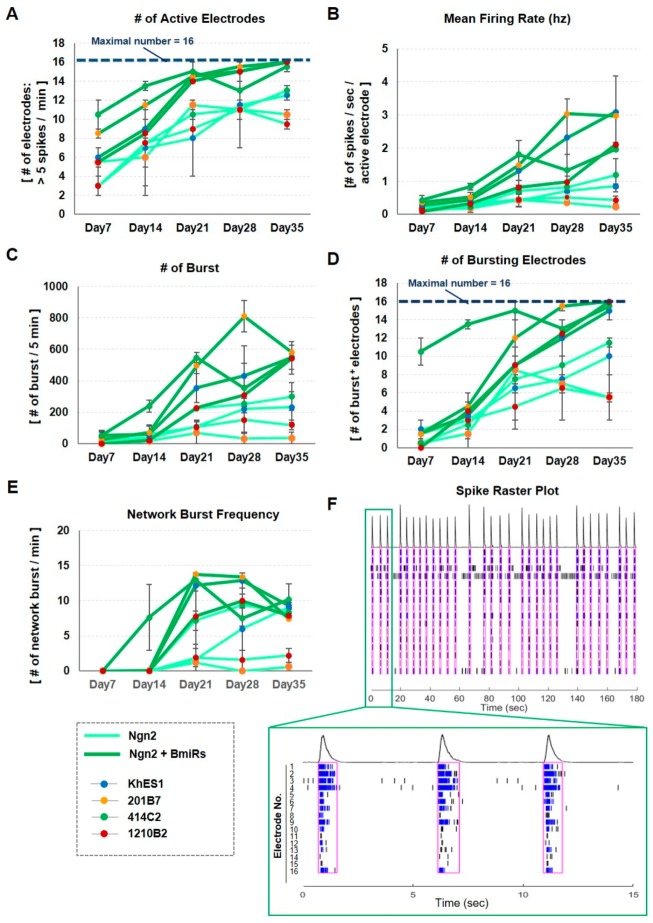
Sequential evaluation of neuronal activity in the neuronal culture using microelectrode array (MEA). The electrophysiological analyses were continuously performed using MEA from day 7 to 35 after neuronal induction. The number of active electrodes (**A**), mean firing rate (**B**), the number of burst (**C**), the number of bursting electrodes (**D**), and network burst frequency (**E**) were calculated using Neural Metric Tool (Axion Biosystems). (n = 4 independent experiments; mean ± SEM) (**F**) A typical 3-minute raster plot drawn with KhES1-derived neurons induced by the overexpression of Ngn2 with BmiRs on day 35. The figure below is an enlargement of the first 15 seconds. The black lines and the blue lines indicate single spikes and spikes defined in a burst, and the time surrounded by magenta indicates the moment when a network burst occurs. See the method section for detailed description of the analysis.

From the results described above, Ngn2 + BmiRs most likely affected neural network activity; hence we proceeded in conducting electrophysiological analysis using a micro/multi-electrode array MEA. Specifically, cells were seeded on a plate in which 16 electrodes were placed per well, and neuronal differentiation was induced as done in the previous experiments. Measurements were performed over time until day 35 (Figure S4A). As a result, in the BmiRs+ group, electrodes showing several active spikes appeared on day 7, and almost all electrodes showed spikes on day 21. Conversely, in the BmiRs- group, the number of spike-positive electrodes slowly increased over time (Figure 5A). The spike frequency (MFR) per electrode from which the spikes appeared was also higher for BmiRs+ on day 21 (Figure 5B). Additionally, when the culture progressed, burst firing which involves the continuous occurrence of spikes were observed (Figure 5F, each line segment is a spike at each electrode, and the blue label indicates burst firing). The number of bursts and the number of electrodes showing bursts were also significantly higher in the “BmiRs+” group (Figure 5C,D). Finally, we examined whether bursts occurred synchronously among the electrodes. Again, BmiRs+ showed significantly higher network burst frequency, but for khES1 and 414C2, Ngn2 alone also induced relatively high network bursts. The area surrounded by magenta in Figure 5E,F indicates the timing of the synchronous burst. This result suggests that in the “BmiRs+” group, neuronal connections were formed within a single well, while the “BmiRs-” group may have had a partial bias in neuronal communication (Figure S4B).

### 3.6. Early Expression of Neuronal Phenotypes of Alzheimer’s Disease (AD) in BmiRs-Induced Neurons

**Figure 6:**
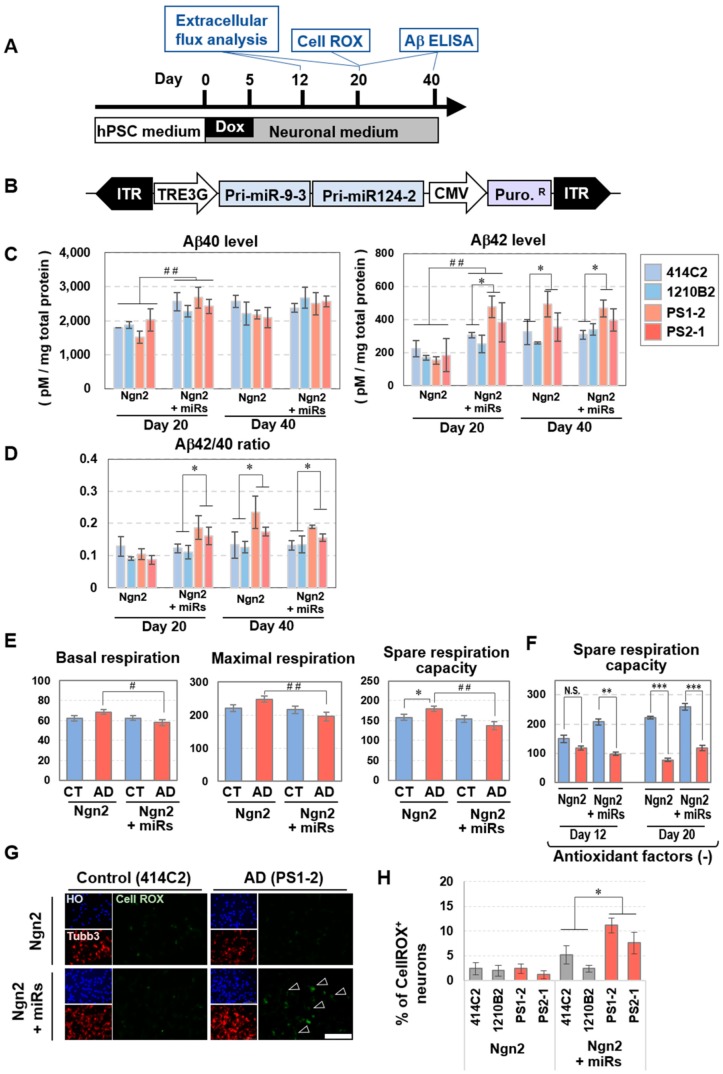
Early phenotype expression of the PS1, PS2 mutant iPS cell-derived neurons by the introduction of miRs. (**A**) Overview of the neuronal culture protocol and phenotype assay. (**B**) Design of the lentiviral vector for Bcl-xL-free miR-9-124-mediated neuronal conversion. The sequences for pri-miR-9-3 and pri-miR-124-2 were included in order to induce the expressions of miR-9 and miR-124, respectively. (**C**) Secreted Aβ40 and Aβ42 levels in the neuronal cell culture derived from the healthy control induced-pluripotent stem cells (iPSCs) and the iPSCs with PSEN1 or 2 mutation at day 20 and day 40. (n = 5 independent experiments; mean ± SEM, ^##^ *p* < 0.01 by Turkey’s test versus CT (miRs-, Day20); * *p* < 0.05, Turkey’s test versus the healthy donor group) (**D**) The ratio of Aβ42/40 (n = 5 independent experiments; mean ± SEM, * *p* < 0.05, Turkey’s test versus the healthy donor group) (**E**,**F**) Functional analysis of mitochondrial respiration in the neurons at day12. Basal oxygen consumption rate (OCR), maximal respiration and spare respiration capacity in healthy control and Alzheimer’s disease (AD) neurons induced by overexpression of Ngn2 with or without miRs in the same neuronal medium used in the experiments above (**E**). Total OCR profile along the assay time course is described in Figure S6B. Spare respiration capacity in a neuronal medium without antioxidant factors are shown in (**F**). (n = 5 independent experiments; mean ± SEM; ^#^ *p* < 0.05; ^##^ *p* < 0.01, Tukey’s test vs. *Ngn2* without miRs, * *p* < 0.05, ** *p* < 0.01, *** *p* < 0.001, Turkey’s test vs. healthy control group) (**G**) Representative images of immunocytochemistry for neurons using CellROX, a marker for reactive oxygen species. Arrowheads indicate CellROX signals. (**H**) CellROX+ cell population in neurons. (n = 4 independent experiments; mean ± SEM; * *p* < 0.05, Turkey’s test vs. healthy control group).

Thus far, we showed that the acquisition of early neuronal maturity was made possible by adding BmiRs during neural induction by Ngn2, which alone was generally thought to result in low neuronal maturation. Therefore, we considered that this differentiation method might be superior to those described in previous reports in terms of a pathological evaluation system for neuronal diseases. For the present study, we decided to examine AD, as it is a highly prevalent disease. Familial AD (FAD) is most frequently caused by mutations in the genes encoding the γ-secretase-component, one of the three components of the amyloid precursor protein (APP) processing pathway, which is encoded by the *presenilin-1* (*PS1*) and *presenilin-2* (*PS2*) genes, or by mutations in the APP gene itself, whereas a growing consensus suggests that sporadic AD (SAD) is more likely caused by impaired clearance of Aβ. We have already established iPS cells from patients with mutations in PS1 and PS2 and have observed a phenotypic expression of high extracellular release of Aβ42 [34,35]. In regard to the method of this study, we determined that overexpression of Bcl-xL would mask the consequences of the stress response to cells, which would not be appropriate for the evaluation of disease phenotypes. Thus, instead of the BmiRs expression used in the previous experiments, we used lentiviral vectors that do not contain the Bcl-xL gene and can express only miR-9/9* and/or miR-124 (Figure 6A,B). As the backbone of the miRs expression vector used for this experiment is different from that in the previous experiments, we reconfirmed the degree to which miR-9/9*, miR-124, miR-9/9*-124, Bcl-xL, and Bcl-xL-miR-9/9*-124, which have been inserted into the new vector backbone, would induce neural differentiation (Figure 6B, Figure S5A). Analysis on day 14 confirmed that neural differentiation can be performed with high accuracy using any of the vectors when added to Ngn2. Without Ngn2, miR-9/9* + miR-124 + Bcl-xL showed the greatest neurite extension, followed by miR-9/9* + miR-124, and then miR-9/9* or miR-124 alone. Bcl-xL alone did not show neurites even in the neuronal medium (Figure S5B). We then introduced TetO-Ngn2 and TetO-Ngn2 + miRs into the PS1 and PS2 mutant cells (Figure S6A).

First, as we have done in several previous reports [34,35], we quantified the amount of Aβ40 and Aβ42 in the cell culture supernatant by ELISA. Consequently, on day 20, the amounts of both Aβ species were increased with the addition of miRs, regardless of whether the cells were healthy or affected with AD, which was almost equivalent to the amount in the miRs- group on day 40. Moreover, possibly due to neuronal maturation, the PS1 and PS2 cells of the miRs- groups showed a tendency of increased Aβ42 levels, which became apparent on day 40. In contrast, the miRs+ group showed similar amounts and increase of Aβ42 levels on both days 20 and 40. The amount of Aβ42 in the miRs+ group did not rise further beyond day 40 (Figure 6C,D).

In addition, as has been reported for many neurodegenerative diseases, mitochondrial dysfunction has also been suggested in AD [36,37,38]. Therefore, we quantified the oxygen consumption rate (OCR) using an extracellular flux analyzer to investigate energy production by mitochondria in this culture method. Just as the increase in the amount of Aβ42 in the culture supernatant was confirmed at an early stage by expression of miRs, phenotypic differences for this experiment was also verified with the shortest possible culture time. Analysis performed on day 12 revealed that basal respiration and maximal respiration slightly decreased in the AD group with the expression of miRs. Conversely, there was no difference between control (CT) and AD groups even with miRs. However, the AD group resulted in slightly higher spare respiration in the absence of miRs (Figure 6E, Figure S6B). As the disease phenotype may not be sufficiently recaptured using the current neuronal medium, after day 5, the cells conditions were changed to use a medium with B27 Supplement whose antioxidant factors had been removed. As a result, in particular, spare respiration was decreased in the AD miRs+ group on day 12, while it did not show a significant decrease in the AD miRs- group on day 12. However, they significantly decreased after day 20 (Figure 6F). These results suggest that mitochondrial dysfunction in AD cells can be expressed at a very rapid stage by optimizing the induction and culture conditions. Finally, whether active oxygen accumulates in the cells was examined using CellROX fluorescence. Results confirmed higher signals in the AD miRs^+^ group (Figure 6G,H, Figure S6C).

**Figure 7:**
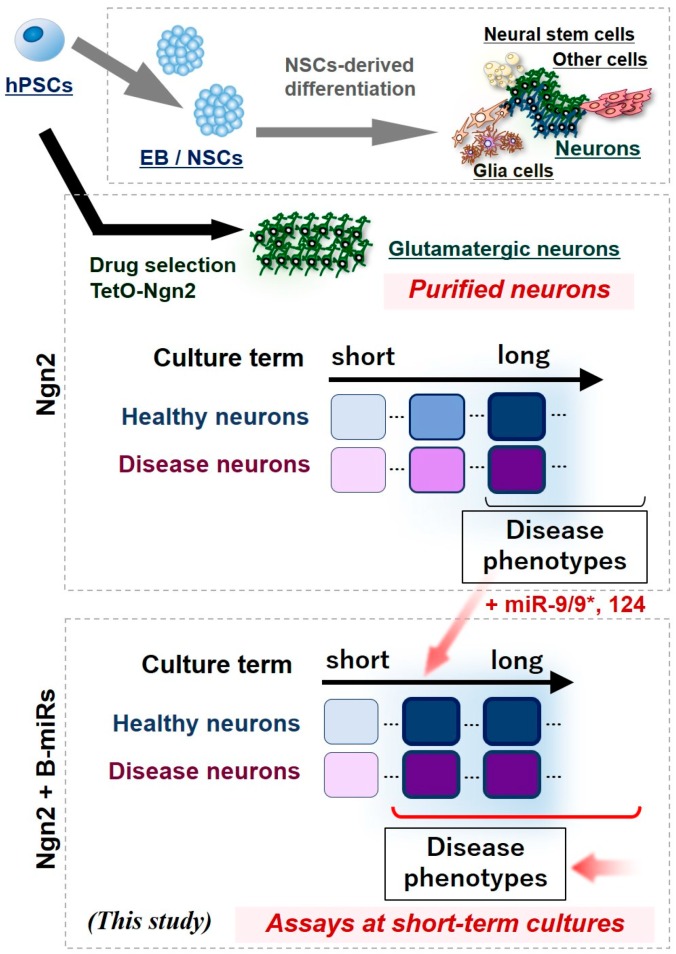
Accelerated pathological analysis and drug discovery screening resulting from early neural differentiation and maturation from pluripotent stem cells by combining the expression of Neurogenin2 and microRNA-9/9*-124. In contrast to the conventional induction method using dual SMAD inhibitors, transient expression of *Ngn2* gene enables a purified population of neurons to be obtained. On the other hand, the Ngn2 method is insufficient in terms of neuronal maturation. Thus, by adding BmiRs expression used in this study, neuronal function can be rapidly enhanced. This enables phenotypic evaluation, which originally required long-term culture, to be performed within short-term culture.

Based on the results above, the addition of miR-9/9* and miR-124 to the Ngn2-induction system is advantageous for performing phenotypic analysis of AD using iPS cells, as rapid maturation can be achieved and consequently, disease phenotypes can be easily detected at early timepoints (Figure 7). This suggests that the induction method in this study is a very useful tool for analyzing neuronal pathophysiology and for drug screening.

## 4. Discussion

In recent years, research on human neural cells using iPSCs has become widespread [3]. In fact, iPSC-based approaches for studying the pathogenesis of neurological diseases, which are difficult to model with animals, are being explored, and research on therapeutic and preventive agents for such diseases has been accelerated. In particular, dementia including AD [39,40,41], Parkinson’s disease [42,43], and ALS [43,44,45,46,47] are among the diseases that are actively studied so that the disease states can be further elucidated and therapeutic agents can be developed for intractable diseases. In terms of neural differentiation methods, the emergence of a neuronal induction method using the transient expression of *Ngn2* greatly enhanced neuronal induction efficiencies [8]. However, functional maturation was considered to be insufficient, even with this method. To overcome this issue, we proposed a new induction system for generating mature neurons using the overexpression of *Ngn2* with miR-9/9*, miR-124 and *Bcl-xL*, which was previously reported as a tool for direct conversion of neurons from human dermal fibroblasts [21,22]. In this study, using hPSCs of various backgrounds and combining these two induction techniques (Ngn2+BmiRs), we succeeded in increasing the rate of differentiation and maturation while maintaining high-purity differentiation into excitatory neurons by overexpression of *Ngn2*. It is particularly noteworthy that spontaneous oscillation of intracellular calcium was observed, and that synaptic protein expression increased and accumulated in neurites. Most likely as a reflection of these phenomena, early firing spikes, bursts, and even synchronous network bursts were also detected in the Ngn2+BmiRs using MEA. These results were possibly significant for the analysis of pathophysiology in neuronal diseases. We used iPSCs from FAD patients [34,35], which were acquired after the disease phenotypes had become apparent in the patients. Ngn2+miRs promoted extracellular secretion of Aβ40 and Aβ42 from early stages of culture, resulting in clearly higher Aβ42/40 ratios in patients with *PS1* and *PS2* mutations. Furthermore, it was possible to detect abnormalities in mitochondrion-mediated cellular respiratory levels and active oxygen accumulation at an early stage. We strongly anticipate that this novel method for neuronal differentiation from PSCs, which enables early maturation as shown in this study, will be useful for analyses of AD and numerous other types of neuronal diseases. Interestingly, however, the data in this study also showed unexpected results and several issues remain to be further examined. For example, Ngn2+BmiRs+ temporarily increased PSC marker genes expression compared to Ngn2+BmiRs-. In the early differentiation stages, the undifferentiated cells died easily, although in principle, the Bcl-xL should support survival in the undifferentiated state. As of present, the details regarding this phenomenon are unknown. As described above, the NSC marker expression levels were also slightly increased in the Ngn2+BmiRs+. This may possibly be explained by the presence of a very rare population of BmiRs+ expressing cells with impaired *Ngn2* expression. Although these cells usually die or differentiate into other lineages, they may have survived because of the Bcl-xL expression and may have forcibly differentiated into NSCs due to the neural medium.

Because we used Bcl-xL for apoptosis inhibition, it may be possible that cells are actively switching their energy production pathway to use oxidative phosphorylation [48,49]. In the future, in order to evaluate the differentiation tendency of the rare populations that existed in our study, it may be required to verify their gene expression on a single-cell basis.

Previous reports have also suggested that neuronal maturation by miR-9, miR-124, and Bcl-xL may be due to accelerated differentiation, and that the differentiated cells may reach maturity through different pathways [50,51,52,53]. Thus, there appears to be room for verification regarding what happens when these factors are continuously expressed for a long period. In this case, excessive miRs expression may alter the intrinsic homeostasis of neurons [54,55,56] masking the disease phenotype. Therefore, disease analysis will need to be carefully performed in consideration of these circumstances.

Further optimization of this current method will deepen our understanding of various neuronal disorders and help elucidate the fundamental neuronal ability of miRNAs. In this study, we focused on forebrain diseases, but as shown in [57], by introducing transcription factors that control various regions and adding compounds [5], a greater variety of neuronal-disease types could be investigated. In fact, as a method for inducing differentiation into motor neurons, efficient differentiation can be performed by adding *Isl1* and *Lhx3* to the BmiRs [15]. Alternatively, introducing *Ctip2*, *Dlx1*, *Dlx2*, and *Myt1l* to the BmiRs promotes the generation of striatal medium spiny neurons [58].

iPSCs reprogrammed from various types of cells such as blood cells have already been established [59,60,61]. Fortunately, this study demonstrated that iPSCs of various backgrounds can be successfully differentiated into neurons. In that regard, the original source of cells may not necessarily need to be PSCs. Direct conversion by expression of transcription factors and miRs was originally performed in human fibroblasts [18,21,62,63] and in recent years, a technique for directly converting neurons from blood cells has also been established [64].

In recent years, brain organoids [65,66,67] and cell transplantation into animal brains [68] have been reported as techniques for enhancing functional maturity in the field of brain research using hPSCs. Compared to these techniques, the high-purity and rapid induction system described in this study has strengths for biochemical analysis and may enable robust disease phenotyping and drug discovery using multiple samples. The next step optimization for future medical research will be to simplify the gene transfer process and minimize the effects of the genomic insertion.

## 5. Conclusions

In this study, we focused on how to achieve neural maturation from PSCs in vitro. Transient expression of the Ngn2 gene, which is used as a method for inducing differentiation of pluripotent stem cells into pure excitable neurons, was insufficient for acquiring neuronal maturation. We used the miR-9/9*, miR-124, and Bcl-xL genes (BmiRs), which have been used in techniques for direct conversion of human dermal fibroblasts to neurons. We demonstrated that the addition of BmiRs to the Ngn2 expression system in PSCs can enhance neuronal maturation of the differentiating cells. In particular, the generated neurons had increased calcium activity and synapse formation. In addition, the MEA analyses showed that the electrical network activity was very high. Using this technique, AD-associated phenotypes could be manifested in a shorter culture period, prompting pathological analysis of various types of neuronal diseases and the development of therapeutic drugs.

## Supplementary Materials

The following are available online at https://www.mdpi.com/2073-4409/9/3/532/s1, Figure S1: Brightfield imaging of neuronal morphogenesis resulting from neuronal media or BmiRs overexpression, Figure S2: Comparison of neuronal differentiation and cell proliferation by combination of Neurogenin2 and BmiRs, Figure S3: The induction strategies for NSCs-derived neurons and cell culture images used for gene expression analyses, Figure S4: Representative Raster Plot image drawn by MEA tool software, Figure S5: Induction of neuronal differentiation by miR9, miR124 or BclxL individually, Figure S6: PS1 and PS2 mutants used in this study and their in vitro pathological evaluation, Table S1: List of primers (related to Figure 1E, Figure 3), Table S2: List of antibodies (related to Figure 1D, Figure 2F,G, Figure 4G,H, Figure 6G, Figure S2B, and Figure S6C).

## Abbreviations

Aβ	amyloid beta
AD	Alzheimer’s disease
APP	amyloid precursor protein
BDNF	brain-derived neurotrophic factor
BmiRs	Bcl-xL, miR-9/9* and miR-124
BSA	bovine serum albumin
dbcAMP	N,2′-O-dibutyryladenosine 3′,5′-phosphoric acid
DMEM	Dulbecco’s modified Eagle medium
Dox	Doxycycline
EBs	embryonic bodies
ELISA	enzyme-linked immunosorbent assay
FAD	familial Alzheimer’s disease
FBS	fetal bovine serum
GDNF	glial cell-derived neurotrophic factor
hESCs	human embryonic stem cells
Ho	Hoechst33342
hiPSCs	human induced pluripotent stem cells
hPSCs	human pluripotent stem cells
IRES	internal ribosome entry site
ITR	inverted terminal repeats
MEA	multiple/micro electrode array
MFR	mean firing rate
mKO1	monomeric Kusabira Orange 1
MOI	multiplicity of infection
NGS	normal goat serum
NGN2	Neurogenin2
NM	culture medium for human neurons
NSCs	neural stem cells
NT-3	neurotrophin-3
OCR	oxygen consumption rates
PBMC	peripheral blood mononuclear cells
PBS	phosphate buffered saline
PFA	Paraformaldehyde
PS1	presenilin-1
PS2	presenilin-2
PSCs	pluripotent stem cells
PSM	culture medium for human pluripotent stem cells
ROS	reactive oxygen species
rtTA	reverse tetracycline transactivator
SAD	sporadic Alzheimer’s disease

## References

[1] Takahashi K., Tanabe K., Ohnuki M., Narita M., Ichisaka T., Tomoda K., Yamanaka S. (2007). Induction of pluripotent stem cells from adult human fibroblasts by defined factors. Cell.

[2] Okano H., Yamanaka S. (2014). iPS cell technologies: Significance and applications to CNS regeneration and disease. Mol. Brain.

[3] Imaizumi Y., Okano H. (2014). Modeling human neurological disorders with induced pluripotent stem cells. J. Neurochem..

[4] Chambers S.M., Fasano C.A., Papapetrou E.P., Tomishima M., Sadelain M., Studer L. (2009). Highly efficient neural conversion of human ES and iPS cells by dual inhibition of SMAD signaling. Nat. Biotechnol..

[5] Imaizumi K., Sone T., Ibata K., Fujimori K., Yuzaki M., Akamatsu W., Okano H. (2015). Controlling the Regional Identity of hPSC-Derived Neurons to Uncover Neuronal Subtype Specificity of Neurological Disease Phenotypes. Stem Cell Rep..

[6] Matsumoto T., Fujimori K., Andoh-Noda T., Ando T., Kuzumaki N., Toyoshima M., Tada H., Imaizumi K., Ishikawa M., Yamaguchi R. (2016). Functional Neurons Generated from T Cell-Derived Induced Pluripotent Stem Cells for Neurological Disease Modeling. Stem Cell Rep..

[7] Fujimori K., Matsumoto T., Kisa F., Hattori N., Okano H., Akamatsu W. (2017). Escape from Pluripotency via Inhibition of TGF-β/BMP and Activation of Wnt Signaling Accelerates Differentiation and Aging in hPSC Progeny Cells. Stem Cell Rep..

[8] Zhang Y., Pak C., Han Y., Ahlenius H., Zhang Z., Chanda S., Marro S., Patzke C., Acuna C., Covy J. (2013). Rapid single-step induction of functional neurons from human pluripotent stem cells. Neuron.

[9] Nakamoto F.K., Okamoto S., Mitsui J., Sone T., Ishikawa M., Yamamoto Y., Kanegae Y., Nakatake Y., Imaizumi K., Ishiura H. (2018). The pathogenesis linked to coenzyme Q10 insufficiency in iPSC-derived neurons from patients with multiple-system atrophy. Sci. Rep..

[10] Ishii T., Ishikawa M., Fujimori K., Maeda T., Kushima I., Arioka Y., Mori D., Nakatake Y., Yamagata B., Nio S. (2019). In vitro Modeling of the Bipolar Disorder and Schizophrenia Using Patient-Derived Induced Pluripotent Stem Cells with Copy Number Variations of PCDH15 and RELN. eNeuro.

[11] Mertens J., Marchetto M.C., Bardy C., Gage F.H. (2016). Evaluating cell reprogramming, differentiation and conversion technologies in neuroscience. Nat. Rev. Neurosci..

[12] Nehme R., Zuccaro E., Ghosh S.D., Li C., Sherwood J.L., Pietilainen O., Barrett L.E., Limone F., Worringer K.A., Kommineni S. (2018). Combining NGN2 Programming with Developmental Patterning Generates Human Excitatory Neurons with NMDAR-Mediated Synaptic Transmission. Cell Rep..

[13] Coolen M., Bally-Cuif L. (2009). MicroRNAs in brain development and physiology. Curr. Opin. Neurobiol..

[14] Lu Y.L., Yoo A.S. (2018). Mechanistic Insights into MicroRNA-Induced Neuronal Reprogramming of Human Adult Fibroblasts. Front. Neurosci..

[15] Abernathy D.G., Kim W.K., McCoy M.J., Lake A.M., Ouwenga R., Lee S.W., Xing X., Li D., Leem H.J., Heuckeroth R.O. (2017). MicroRNAs Induce a Permissive Chromatin Environment that Enables Neuronal Subtype-Specific Reprogramming of Adult Human Fibroblasts. Cell Stem Cell.

[16] Lee S.W., Oh Y.M., Lu Y.L., Kim W.K., Yoo A.S. (2018). MicroRNAs Overcome Cell Fate Barrier by Reducing EZH2-Controlled REST Stability during Neuronal Conversion of Human Adult Fibroblasts. Dev. Cell.

[17] Lessard J., Wu J.I., Ranish J.A., Wan M., Winslow M.M., Staahl B.T., Wu H., Aebersold R., Graef I.A., Crabtree G.R. (2007). An essential switch in subunit composition of a chromatin remodeling complex during neural development. Neuron.

[18] Yoo A.S., Staahl B.T., Chen L., Crabtree G.R. (2009). MicroRNA-mediated switching of chromatin-remodelling complexes in neural development. Nature.

[19] Wu J.I., Lessard J., Olave I.A., Qiu Z., Ghosh A., Graef I.A., Crabtree G.R. (2007). Regulation of dendritic development by neuron-specific chromatin remodeling complexes. Neuron.

[20] Staahl B.T., Crabtree G.R. (2013). Creating a neural specific chromatin landscape by npBAF and nBAF complexes. Curr. Opin. Neurobiol..

[21] Yoo A.S., Sun A.X., Li L., Shcheglovitov A., Portmann T., Li Y., Lee-Messer C., Dolmetsch R.E., Tsien R.W., Crabtree G.R. (2011). MicroRNA-mediated conversion of human fibroblasts to neurons. Nature.

[22] Richner M., Victor M.B., Liu Y., Abernathy D., Yoo A.S. (2015). MicroRNA-based conversion of human fibroblasts into striatal medium spiny neurons. Nat. Protoc..

[23] Maryanovich M., Gros A. (2013). A ROS rheostat for cell fate regulation. Trends Cell Biol..

[24] Victor M.B., Richner M., Hermanstyne T.O., Ransdell J.L., Sobieski C., Deng P.Y., Klyachko V.A., Nerbonne J.M., Yoo A.S. (2014). Generation of human striatal neurons by microRNA-dependent direct conversion of fibroblasts. Neuron.

[25] Suemori H., Yasuchika K., Hasegawa K., Fujioka T., Tsuneyoshi N., Nakatsuji N. (2006). Efficient establishment of human embryonic stem cell lines and long-term maintenance with stable karyotype by enzymatic bulk passage. Biochem. Biophys. Res. Commun..

[26] Nakagawa M., Taniguchi Y., Senda S., Takizawa N., Ichisaka T., Asano K., Morizane A., Doi D., Takahashi J., Nishizawa M. (2014). A novel efficient feeder-free culture system for the derivation of human induced pluripotent stem cells. Sci. Rep..

[27] Okita K., Matsumura Y., Sato Y., Okada A., Morizane A., Okamoto S., Hong H., Nakagawa M., Tanabe K., Tezuka K. (2011). A more efficient method to generate integration-free human iPS cells. Nat. Methods.

[28] Miyazaki T., Isobe T., Nakatsuji N., Suemori H. (2017). Efficient Adhesion Culture of Human Pluripotent Stem Cells Using Laminin Fragments in an Uncoated Manner. Sci. Rep..

[29] Matsushita M., Nakatake Y., Arai I., Ibata K., Kohda K., Goparaju S.K., Murakami M., Sakota M., Chikazawa-Nohtomi N., Ko S.B.H. (2017). Neural differentiation of human embryonic stem cells induced by the transgene-mediated overexpression of single transcription factors. Biochem. Biophys. Res. Commun..

[30] Sone T., Imamoto F. (2012). Methods for constructing clones for protein expression in mammalian cells. Methods Mol. Biol..

[31] Iida T., Iwanami A., Sanosaka T., Kohyama J., Miyoshi H., Nagoshi N., Kashiwagi R., Toyama Y., Matsumoto M., Nakamura M. (2017). Whole-Genome DNA Methylation Analyses Revealed Epigenetic Instability in Tumorigenic Human iPS Cell-Derived Neural Stem/Progenitor Cells. Stem Cells.

[32] Magnani E., Bartling L., Hake S. (2006). From Gateway to MultiSite Gateway in one recombination event. BMC Mol. Biol..

[33] Sakaue-Sawano A., Kurokawa H., Morimura T., Hanyu A., Hama H., Osawa H., Kashiwagi S., Fukami K., Miyata T., Miyoshi H. (2008). Visualizing spatiotemporal dynamics of multicellular cell-cycle progression. Cell.

[34] Yagi T., Ito D., Okada Y., Akamatsu W., Nihei Y., Yoshizaki T., Yamanaka S., Okano H., Suzuki N. (2011). Modeling familial Alzheimer’s disease with induced pluripotent stem cells. Hum. Mol. Genet..

[35] Sho M., Ichiyanagi N., Imaizumi K., Ishikawa M., Morimoto S., Watanabe H., Okano H. (2019). A combinational treatment of carotenoids decreases Aβ secretion in human neurons via β-secretase inhibition. Neurosci. Res..

[36] Wilkins J.M., Trushina E. (2018). Application of Metabolomics in Alzheimer’s Disease. Front. Neurol..

[37] Flannery P.J., Trushina E. (2019). Mitochondrial dynamics and transport in Alzheimer’s disease. Mol. Cell Neurosci..

[38] Cadonic C., Sabbir M.G., Albensi B.C. (2016). Mechanisms of Mitochondrial Dysfunction in Alzheimer’s Disease. Mol. Neurobiol..

[39] Kondo T., Asai M., Tsukita K., Kutoku Y., Ohsawa Y., Sunada Y., Imamura K., Egawa N., Yahata N., Okita K. (2013). Modeling Alzheimer’s disease with iPSCs reveals stress phenotypes associated with intracellular Aβ and differential drug responsiveness. Cell Stem Cell.

[40] Imamura K., Sahara N., Kanaan N.M., Tsukita K., Kondo T., Kutoku Y., Ohsawa Y., Sunada Y., Kawakami K., Hotta A. (2016). Calcium dysregulation contributes to neurodegeneration in FTLD patient iPSC-derived neurons. Sci. Rep..

[41] Nakamura M., Shiozawa S., Tsuboi D., Amano M., Watanabe H., Maeda S., Kimura T., Yoshimatsu S., Kisa F., Karch C.M. (2019). Pathological Progression Induced by the Frontotemporal Dementia-Associated R406W Tau Mutation in Patient-Derived iPSCs. Stem Cell Rep..

[42] Imaizumi Y., Okada Y., Akamatsu W., Koike M., Kuzumaki N., Hayakawa H., Nihira T., Kobayashi T., Ohyama M., Sato S. (2012). Mitochondrial dysfunction associated with increased oxidative stress and α-synuclein accumulation in PARK2 iPSC-derived neurons and postmortem brain tissue. Mol. Brain.

[43] Tabata Y., Imaizumi Y., Sugawara M., Andoh-Noda T., Banno S., Chai M., Sone T., Yamazaki K., Ito M., Tsukahara K. (2018). T-type Calcium Channels Determine the Vulnerability of Dopaminergic Neurons to Mitochondrial Stress in Familial Parkinson Disease. Stem Cell Rep..

[44] Ichiyanagi N., Fujimori K., Yano M., Ishihara-Fujisaki C., Sone T., Akiyama T., Okada Y., Akamatsu W., Matsumoto T., Ishikawa M. (2016). Establishment of In Vitro FUS-Associated Familial Amyotrophic Lateral Sclerosis Model Using Human Induced Pluripotent Stem Cells. Stem Cell Rep..

[45] Fujimori K., Ishikawa M., Otomo A., Atsuta N., Nakamura R., Akiyama T., Hadano S., Aoki M., Saya H., Sobue G. (2018). Modeling sporadic ALS in iPSC-derived motor neurons identifies a potential therapeutic agent. Nat. Med..

[46] Akiyama T., Suzuki N., Ishikawa M., Fujimori K., Sone T., Kawada J., Funayama R., Fujishima F., Mitsuzawa S., Ikeda K. (2019). Aberrant axon branching via Fos-B dysregulation in FUS-ALS motor neurons. EBioMedicine.

[47] Imamura K., Izumi Y., Watanabe A., Tsukita K., Woltjen K., Yamamoto T., Hotta A., Kondo T., Kitaoka S., Ohta A. (2017). The Src/c-Abl pathway is a potential therapeutic target in amyotrophic lateral sclerosis. Sci. Transl. Med..

[48] Berninger B., Costa M.R., Koch U., Schroeder T., Sutor B., Grothe B., Götz M. (2007). Functional properties of neurons derived from in vitro reprogrammed postnatal astroglia. J. Neurosci..

[49] Gascón S., Murenu E., Masserdotti G., Ortega F., Russo G.L., Petrik D., Deshpande A., Heinrich C., Karow M., Robertson S.P. (2016). Identification and Successful Negotiation of a Metabolic Checkpoint in Direct Neuronal Reprogramming. Cell Stem Cell.

[50] Sim S.E., Lim C.S., Kim J.I., Seo D., Chun H., Yu N.K., Lee J., Kang S.J., Ko H.G., Choi J.H. (2016). The Brain-Enriched MicroRNA miR-9-3p Regulates Synaptic Plasticity and Memory. J. Neurosci..

[51] Giusti S.A., Vogl A.M., Brockmann M.M., Vercelli C.A., Rein M.L., Trümbach D., Wurst W., Cazalla D., Stein V., Deussing J.M. (2014). MicroRNA-9 controls dendritic development by targeting REST. Elife.

[52] Dajas-Bailador F., Bonev B., Garcez P., Stanley P., Guillemot F., Papalopulu N. (2012). microRNA-9 regulates axon extension and branching by targeting Map1b in mouse cortical neurons. Nat. Neurosci..

[53] Hou Q., Ruan H., Gilbert J., Wang G., Ma Q., Yao W.D., Man H.Y. (2015). MicroRNA miR124 is required for the expression of homeostatic synaptic plasticity. Nat. Commun..

[54] Topol A., Zhu S., Hartley B.J., English J., Hauberg M.E., Tran N., Rittenhouse C.A., Simone A., Ruderfer D.M., Johnson J. (2016). Dysregulation of miRNA-9 in a Subset of Schizophrenia Patient-Derived Neural Progenitor Cells. Cell Rep..

[55] Higuchi F., Uchida S., Yamagata H., Abe-Higuchi N., Hobara T., Hara K., Kobayashi A., Shintaku T., Itoh Y., Suzuki T. (2016). Hippocampal MicroRNA-124 Enhances Chronic Stress Resilience in Mice. J. Neurosci..

[56] Madelaine R., Sloan S.A., Huber N., Notwell J.H., Leung L.C., Skariah G., Halluin C., Paşca S.P., Bejerano G., Krasnow M.A. (2017). MicroRNA-9 Couples Brain Neurogenesis and Angiogenesis. Cell Rep..

[57] Tsunemoto R., Lee S., Szűcs A., Chubukov P., Sokolova I., Blanchard J.W., Eade K.T., Bruggemann J., Wu C., Torkamani A. (2018). Diverse reprogramming codes for neuronal identity. Nature.

[58] Victor M.B., Richner M., Olsen H.E., Lee S.W., Monteys A.M., Ma C., Huh C.J., Zhang B., Davidson B., Yang X.W. (2018). Striatal neurons directly converted from Huntington’s disease patient fibroblasts recapitulate age-associated disease phenotypes. Nat. Neurosci..

[59] Seki T., Yuasa S., Oda M., Egashira T., Yae K., Kusumoto D., Nakata H., Tohyama S., Hashimoto H., Kodaira M. (2010). Generation of induced pluripotent stem cells from human terminally differentiated circulating T cells. Cell Stem Cell.

[60] Fujimori K., Tezuka T., Ishiura H., Mitsui J., Doi K., Yoshimura J., Tada H., Matsumoto T., Isoda M., Hashimoto R. (2016). Modeling neurological diseases with induced pluripotent cells reprogrammed from immortalized lymphoblastoid cell lines. Mol. Brain.

[61] Nakazawa T., Kikuch M., Ishikawa M., Yamamori H., Nagayasu K., Matsumoto T., Fujimoto M., Yasuda Y., Fujiwara M., Okada S. (2017). Differential gene expression profiles in neurons generated from lymphoblastoid B-cell line-derived iPS cells from monozygotic twin cases with treatment-resistant schizophrenia and discordant responses to clozapine. Schizophr. Res..

[62] Pang Z.P., Yang N., Vierbuchen T., Ostermeier A., Fuentes D.R., Yang T.Q., Citri A., Sebastiano V., Marro S., Südhof T.C. (2011). Induction of human neuronal cells by defined transcription factors. Nature.

[63] Vierbuchen T., Ostermeier A., Pang Z.P., Kokubu Y., Südhof T.C., Wernig M. (2010). Direct conversion of fibroblasts to functional neurons by defined factors. Nature.

[64] Tanabe K., Ang C.E., Chanda S., Olmos V.H., Haag D., Levinson D.F., Südhof T.C., Wernig M. (2018). Transdifferentiation of human adult peripheral blood T cells into neurons. Proc. Natl. Acad. Sci. USA.

[65] Lancaster M.A., Renner M., Martin C.A., Wenzel D., Bicknell L.S., Hurles M.E., Homfray T., Penninger J.M., Jackson A.P., Knoblich J.A. (2013). Cerebral organoids model human brain development and microcephaly. Nature.

[66] Kadoshima T., Sakaguchi H., Nakano T., Soen M., Ando S., Eiraku M., Sasai Y. (2013). Self-organization of axial polarity, inside-out layer pattern, and species-specific progenitor dynamics in human ES cell-derived neocortex. Proc. Natl. Acad. Sci. USA.

[67] Birey F., Andersen J., Makinson C.D., Islam S., Wei W., Huber N., Fan H.C., Metzler K.R.C., Panagiotakos G., Thom N. (2017). Assembly of functionally integrated human forebrain spheroids. Nature.

[68] Mansour A.A., Gonçalves J.T., Bloyd C.W., Li H., Fernandes S., Quang D., Johnston S., Parylak S.L., Jin X., Gage F.H. (2018). An in vivo model of functional and vascularized human brain organoids. Nat. Biotechnol..

